# Gut microbiota diversity is prognostic and associated with benefit from chemo‐immunotherapy in metastatic triple‐negative breast cancer

**DOI:** 10.1002/1878-0261.13760

**Published:** 2024-11-15

**Authors:** Andreas Ullern, Kristian Holm, Andreas Hagen Røssevold, Nikolai Kragøe Andresen, Corinna Bang, Ole Christian Lingjærde, Bjørn Naume, Johannes R. Hov, Jon Amund Kyte

**Affiliations:** ^1^ Department of Clinical Cancer Research Oslo University Hospital Norway; ^2^ Department of Cancer Immunology, Institute for Cancer Research Oslo University Hospital Norway; ^3^ Institute of Clinical Medicine University of Oslo Norway; ^4^ Research Institute of Internal Medicine Oslo University Hospital Rikshospitalet Norway; ^5^ Norwegian PSC Research Center, Department of Transplantation Medicine Oslo University Hospital Norway; ^6^ Institute of Clinical Molecular Biology Christian‐Albrechts‐University of Kiel Germany; ^7^ Department of Cancer Genetics, Institute for Cancer Research Oslo University Hospital Norway; ^8^ Center for Bioinformatics, Department of Informatics University of Oslo Norway; ^9^ Department of Oncology Oslo University Hospital Norway; ^10^ Section of Gastroenterology, Department of Transplantation Medicine Oslo University Hospital Norway; ^11^ Faculty of Health Sciences Oslo Metropolitan University Norway

**Keywords:** alpha diversity, biomarker, gut microbiota, immunotherapy, triple‐negative breast cancer

## Abstract

The gut microbiota influences multiple aspects of human health and disease. Several studies have indicated an association between the gut microbiota and response to immune checkpoint inhibitors in various cancers, but there is scarce data from breast cancer. The randomized ALICE trial demonstrated improved progression‐free survival (PFS) from adding the programmed cell death 1 ligand 1 (PD‐L1) inhibitor atezolizumab (atezo) to immunomodulating chemotherapy (chemo) in metastatic triple‐negative breast cancer (mTNBC), even for PD‐L1^negative^ disease. Herein, we investigated the microbiota composition and dynamics in the ALICE patients and their association with clinical outcome, by analyzing fecal samples collected at baseline and after 8 weeks. We applied 16S (V3‐V4) rRNA sequencing to characterize the diversity and taxonomic composition. Kaplan–Meier and Cox proportional hazard models were used for time‐to‐event analyses. We found that high alpha diversity by Faith's phylogenetic diversity (PD) at baseline was associated with prolonged PFS in the total study population and in the atezo‐chemo arm, but not in the placebo‐chemo arm. Moreover, Faith's PD appeared to be predictive of benefit from atezolizumab. Patients with high Faith's PD exhibited a PFS hazard ratio of 0.34 (*P* = 0.018) in favor of the atezo‐chemo arm, compared to 0.83 (*P* = 0.62) in the low Faith's PD group. Faith's PD was significantly reduced during treatment. At baseline, *Bifidobacterium* was significantly overrepresented in patients without clinical benefit in the atezo‐chemo arm, but not in the placebo‐chemo arm. These findings suggest that alpha diversity by Faith's PD should be further investigated as a prognostic and predictive biomarker in patients with mTNBC receiving chemo‐immunotherapy.

## Graphical abstract

We assessed the associations between the gut microbiota and outcome in metastatic triple‐negative breast cancer patients treated with chemotherapy alone or chemotherapy in combination with immunotherapy. Our data indicate that high gut microbiota alpha diversity was associated with improved clinical outcome and with benefit from immunotherapy.

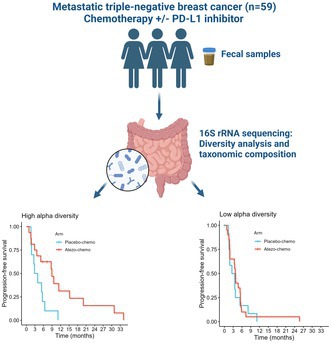

**Abbreviations**
ANCOM‐BC: analysis of compositions of microbiomes with bias correction
ASV: amplicon sequence variant
Atezo: atezolizumab
Chemo: chemotherapy
Faith's PD: Faith's phylogenetic diversity
ICI: immune checkpoint inhibitor
IrAE: immune‐related adverse event
PD‐L1: programmed cell death 1 ligand 1
PFS: progression‐free survival
TNBC: triple‐negative breast cancer

## 1 Introduction

The gut microbiota is considered to influence health and disease. Accumulating evidence is linking the taxonomic composition and diversity of the intestinal flora to outcomes of different anti‐cancer treatments [1]. In multiple cancer types, gut microbiota composition has been associated with response and adverse effects of immune checkpoint inhibitors (ICIs) [2, 3, 4]. However, there is limited consensus on microbial taxa associated with outcome across different studies [5]. Multiple lines of evidence also indicate that the gut microbiota is important for response to chemotherapy [1]. Yet, there is limited data on how the gut microbiota influences patients with breast cancer. Some studies have compared the gut microbiota in breast cancer patients to healthy controls [5, 6, 7]. Further, the gut microbiota in non‐metastatic breast cancer patients undergoing neoadjuvant and adjuvant chemotherapy has been investigated [8, 9, 10, 11]. In addition, gut microbiota profiling has been performed in a small cohort of patients receiving neoadjuvant trastuzumab‐based chemotherapy [12]. However, the impact of the gut microbiota on response and toxicity to ICIs in breast cancer has not been reported.

ICIs targeting PD‐1/PD‐L1 are now approved in combination with chemotherapy in PD‐L1‐positive metastatic triple‐negative breast cancer (mTNBC). Pembrolizumab is approved in the neoadjuvant setting for high‐risk, early‐stage triple‐negative breast cancer, regardless of PD‐L1 status. Unfortunately, many patients do not benefit from the addition of ICIs and some experience immune‐related adverse events (irAEs). Therefore, identifying biomarkers for patient selection is an important unmet need in breast cancer immuno‐oncology [13]. Here, we report the results of the gut microbiota profiling from the randomized, double‐blind, placebo‐controlled phase 2b ALICE trial [14, 15]. This trial evaluated the safety and efficacy of adding the PD‐L1 inhibitor atezolizumab to selected immunomodulatory chemotherapy in mTNBC. In ALICE, this treatment combination gave improved progression‐free survival (PFS) regardless of PD‐L1 status. The trial employed anthracyclines and low‐dose cyclophosphamide, intended to induce immunogenic cell death and counter regulatory T cells, and was the first to indicate clinical benefit for PD‐L1‐negative mTNBC. As observed in other cancer forms, the tail of the PFS curve implied durable benefit from PD‐L1 blockade for a subpopulation of patients.

In the present study, we investigated the associations between gut microbiota and benefit from chemotherapy alone or chemotherapy in combination with PD‐L1 blockade in the ALICE trial. We further explored the relationship between gut microbes and irAE occurrence, and the longitudinal dynamics of the gut microbiota during treatment.

## 2 Materials and methods

### 2.1 Study design and participants

All patients were participants in the randomized, double‐blind, placebo‐controlled phase 2b ALICE trial (NCT03164993). In ALICE, patients with mTNBC were randomized 2:3 to receive chemotherapy alone (placebo‐chemo; *n* = 28) or combined with atezolizumab (atezo‐chemo; *n* = 42). The chemotherapy treatment comprised pegylated liposomal doxorubicin (PLD) 20 mg·m^−2^ i.v. on day 1 of each 14‐day cycle and cyclophosphamide 50 mg p.o. daily in every other 14‐day cycle and was identical in the two arms. Atezolizumab (840 mg) or placebo was given i.v. on day 1 of each cycle.

Between 24 August 2017 and 21 December 2021, patients were enrolled in the study in five centers in Denmark and Norway (Oslo University Hospital, Stavanger University Hospital, St. Olavs University Hospital (Trondheim), Vejle Hospital and Rigshospitalet (Copenhagen)). Eligible patients had metastatic or incurable locally advanced, histologically documented TNBC (negative for human epidermal growth factor receptor 2 [HER2], estrogen receptor [ER] and progesterone receptor [PR]), were 18 years or older, had measurable disease by Immunotherapy Response Evaluation Criteria in Solid Tumors (iRECIST), Eastern Cooperative Oncology Group (ECOG) performance status 0–1 and received maximum one previous line of chemotherapy for metastatic disease. The study was approved by the Regional Committee for Medical Research Ethics South‐East Norway (EC ID: 14195), the Research Ethics Committee in Denmark (EC ID: H‐18018750), the Norwegian Medical Agency (ID: 16/11993), the Danish Medicines Agency (ID: 2018051636) and institutional review boards. All patients gave written informed consent before enrollment and the trial was conducted in adherence to the Declaration of Helsinki.

In the ALICE trial, the primary endpoints were safety and a descriptive assessment of clinical response by progression‐free survival (PFS) in the two arms. Overall survival (OS) and clinical benefit rate (CBR) were among the secondary efficacy endpoints. In the microbiota analyses, only patients who provided fecal samples at baseline, received any study drug and were evaluable by iRECIST were included.

### 2.2 Sample collection

Fecal samples were collected at baseline (before start of study treatment) and after 8 weeks (week 9). Fecal samples were collected in 15 mL sterile containers and stored by the patients at 4 °C, then frozen at −80 °C within 24 h.

### 2.3 Fecal DNA extraction and 16S sequencing

DNA was extracted using the QIAamp Fast DNA stool mini kit automated on the QIAcube. Approximately 200 mg stool was transferred to 0.70 mm Power Bead tubes filled with 1.1 mL ASL lysis buffer (containing Proteinase K). Subsequently, bead beating was performed using SpeedMill PLUS for 45 s at 50 Hz. Samples were then heated to 95 °C for 5 min. DNA binds specifically to QIAamp silica‐gel membrane while contaminants pass through, and PCR inhibitors were removed by an optimized buffer. Variable regions V3 and V4 of the 16S rRNA gene were amplified using the primer pair 357F‐806R in a dual‐barcoding approach, according to Kozich et al. [16] 3 μL of DNA was used for amplification. PCR products were verified using electrophoresis in agarose gel. PCR products were normalized using the SequalPrep Normalization Plate Kit (Thermo Fischer Scientific, Waltham, MA, USA), pooled equimolarly and sequenced on the Illumina MiSeq v3 2 × 300 bp (Illumina Inc., San Diego, CA, USA).

### 2.4 Sequence processing and bioinformatics

Demultiplexing after sequencing was based on 0 mismatches in the barcode sequences. Paired‐end reads were trimmed for primers with cutadapt version 4.0 [17] (parameters: ‐e 0.1 ‐u 1 ‐U 1 ‐‐discard‐untrimmed ‐m 250) and subsequently quality trimmed and merged using bbmerge version 38.90 [18] (parameters: qtrim=r trimq=15 maxlength=500 mininsert=350). The merged contigs were trimmed to 400 bp and denoised to amplicon sequence variants (ASVs) with deblur [19] in qiime2 version 2022.2 [20]. Taxonomic classification of ASVs was done based on RESCRIPT [21] in qiime2 using a naïve Bayes classifier [22] trained on the V3‐V4 region of a preclustered version (99% sequence similarity) of the Silva database version 138 [23].

ASVs from mitochondria, chloroplast or lacking taxonomic annotation on order level were removed and a de‐novo phylogenetic tree was built in qiime2 based on the remaining ASVs. To reduce the effect of uneven sequencing depths, samples were rarefied (subsampled without replacement) to an even level of 6411 counts per sample and all analyses except differential abundance were performed on this rarefied dataset. Alpha diversity metrics (Faith's phylogenetic diversity (PD) and observed ASVs) were calculated in qiime2. Similar bioinformatics methods have been reported in Karlsen et al. [24].

### 2.5 Statistical analyses

PFS, clinical benefit and immune‐related adverse events (irAEs) were selected as clinical endpoints in the microbiota analysis. PFS was defined as the time from randomization until objective disease progression or death. Patients with no events were censored at the date of the last tumor assessment. The clinical benefit rate was defined as the proportion of patients with an objective response according to iRECIST or with stable disease lasting at least until the radiological evaluation at 24 weeks ±7 days. Adverse events were graded using the Common Terminology Criteria for Adverse Events (CTCAE) version 4.0.

Survival curves were estimated using the Kaplan–Meier method and compared using the log‐rank method. The reverse Kaplan–Meier method was used to estimate the median follow‐up time, by censoring for overall survival as the event. Hazard ratios (HR) with 95% confidence intervals (CI) were calculated using the Cox proportional hazards model. Alpha diversity and several clinical factors were analyzed by the univariate and multivariate Cox proportional hazards model for PFS. Assumptions for the Cox models were tested. Variables with *P* < 0.1 in the univariate analysis were selected for the multivariate analysis. R packages survival [25] and survminer [26] were used for time‐to‐event analyses.

The alpha diversity metric Faith's PD was evaluated as both a continuous variable and a binary variable. Both the median value and an optimal, fine‐tuned cut‐off value were tested when Faith's PD was analyzed as a binary variable. To define the optimal cut‐off value for Faith's PD, we constructed an algorithm based on confidence level and group size (minimum 20% in each group). A table was constructed using the log‐rank method for PFS testing all cut‐offs of Faith's PD in the atezo‐chemo arm. To visualize the optimized cut‐off for Faith's PD, a receiver operating characteristic (ROC) curve was built with the pROC package in R [27], with PFS (dichotomized by the median value) as a response variable and Faith's PD as a predictor variable.

Differential abundance testing with ANCOM‐BC2 [28] was done on a prevalence‐filtered (10%) version of the non‐rarefied dataset. Faith's PD was compared between groups using a *t* test or a Wilcoxon rank‐sum test, depending on the distribution. A Wilcoxon signed‐rank test was used for pairwise comparisons in the longitudinal analysis of alpha diversity and taxa‐specific changes between baseline and week 9 (20% prevalence‐filtered). The Kendall rank correlation coefficient was used for correlation analysis. All *P* values given are two‐tailed. Analyses and plots were done in R [29] using packages phyloseq [30], qiime2r [31] and ggplot2 [32].

## 3 Results

### 3.1 Patient characteristics and clinical outcome

**FIGURE 1:**
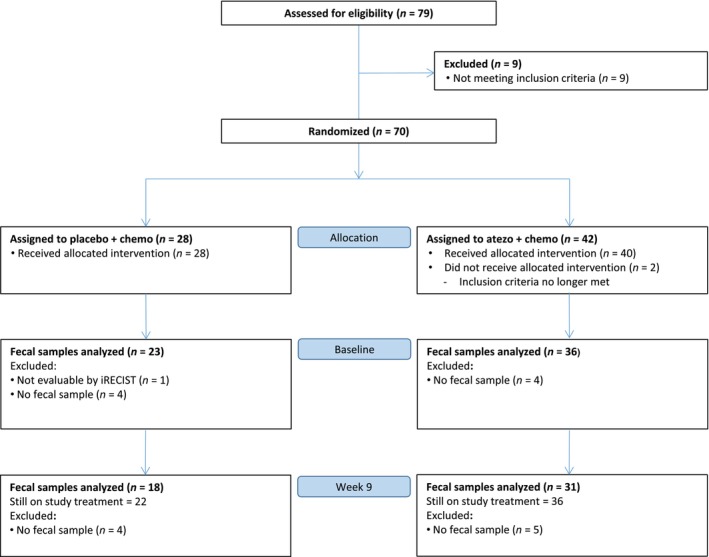
Patient enrollment and stool collection in the ALICE trial. Fecal samples were collected at baseline (before treatment initiation) and week 9 (after 8 weeks of treatment) in the placebo plus chemotherapy arm (placebo + chemo) and in the atezolizumab plus chemotherapy arm (atezo + chemo).

A total of 70 patients were randomized in the ALICE trial. Twenty‐eight patients received treatment in the placebo‐chemo arm and 40 patients received treatment in the atezo‐chemo arm. In the placebo‐chemo arm, 23 patients (82%) delivered fecal samples at baseline for profiling, while 18 (82%) of the 22 patients who remained in the study until week 9 provided paired samples (baseline + week 9). In the atezo‐chemo arm, fecal samples were analyzed from 36 patients (90%) at baseline, whereas 31 (86%) of the 36 patients who continued in the study until week 9 provided paired samples (Figure 1). Median follow‐up time was 32.2 months among patients delivering fecal samples.

Baseline characteristics (for all patients included in the analyses) are summarized in Table S1. The two treatment arms were generally balanced. Of note, we observed no significant difference in alpha diversity measures between the two treatment arms (data not shown). For patients who provided fecal samples at baseline, an improvement in PFS (primary trial endpoint) was observed in the atezo‐chemo arm (HR 0.55, 95% CI 0.32–0.98; Figure S1), which is in line with the results reported for the total study population [15]. Clinical benefit, as defined in the study protocol (objective response or stable disease at 24‐week evaluation), was observed in 56% (20/36) of patients in the atezo‐chemo arm, compared to 35% (8/23) in the placebo‐chemo arm. The clinical benefit rate (secondary trial endpoint) was thus also similar to the entire ALICE study population [15].

### 3.2 Baseline alpha diversity is associated with progression free survival

**FIGURE 2:**
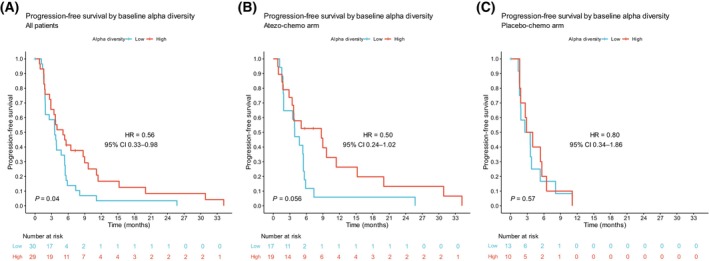
Progression‐free survival by baseline Faith's phylogenetic diversity. Kaplan–Meier plot in (A) all patients, (B) the atezo‐chemo and (C) the placebo‐chemo arm. Patients were classified into low and high diversity groups based on the median score of Faith's phylogenetic diversity. Hazard ratios and 95% confidence intervals were calculated using the Cox proportional hazards model. *P* values were calculated by the log‐rank method.

A diverse composition of the gut microbiota, denoted as high alpha diversity, is considered a possible positive biomarker in immuno‐oncology [5]. The clinical outcome, PD‐L1‐status and alpha diversity measurements for each patient are listed in Table S2. We first investigated the association between baseline alpha diversity and PFS, which was the primary efficacy outcome in the ALICE trial. The patients were classified into low and high alpha diversity groups based on the median scores of alpha diversity. We found that patients with high Faith's PD had significantly prolonged PFS compared to patients with low Faith's PD (HR 0.56, 95% CI 0.33–0.98; *P* = 0.04; Figure 2A). When assessing the two treatment arms separately, the association was preserved in the atezo‐chemo arm (HR 0.50, 95% CI 0.24–1.02; *P* = 0.056; Figure 2B), but not in the placebo‐chemo arm (HR 0.80, 95% CI 0.34–1.86; *P* = 0.57; Figure 2C). Similar analyses were performed using a different alpha diversity metric, observed ASVs, which also indicated prolonged PFS for patients with above median diversity, although not statistically significant (HR 0.68; *P* = 0.15; Figure S2a–c). We also evaluated the relationship between Faith's PD as a continuous variable and PFS. Patients with PFS >6 months had significantly higher Faith's PD compared to those with PFS ≤6 months, in all patients and in the atezo‐chemo arm (Figure S3a–c).

**FIGURE 3:**
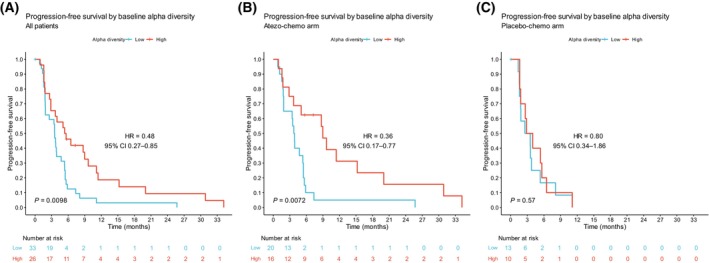
Progression‐free survival by baseline Faith's phylogenetic diversity‐optimized cut‐off. Kaplan–Meier plot in (A) all patients, (B) the atezo‐chemo and (C) the placebo‐chemo arm. Patients were classified into low and high diversity groups based on the optimized cut‐off score of Faith's phylogenetic diversity. Hazard ratios and 95% confidence intervals were calculated using the Cox proportional hazards model. *P* values were calculated by the log‐rank method.

Next, we estimated an optimal cut‐off value for alpha diversity (Faith's PD), with regard to its association with PFS in the atezo‐chemo arm. To this aim, we assessed the confidence level for cut‐offs whereby a minimum of 20% of patients were classified into each group. *P* values for different cut‐offs were estimated by the log‐rank method, and the cut‐off with the lowest *P* value was chosen (Figure S4a). This method produced a cut‐off set at the 56th percentile for Faith's PD by which 26/59 (44%) patients were classified into the high alpha diversity group. To visualize the impact of different cut‐offs, a receiver operating characteristic (ROC) curve was built. The suggested cut‐off from the ROC curve corresponded to the same percentile of Faith PD (Figure S4b). Employing this optimized cut‐off, we found that a high baseline Faith's PD was associated with prolonged PFS in all patients (HR 0.48, 95% CI 0.27–0.85; *P =* 0.0098) and in the atezo‐chemo arm (HR 0.36, 95% CI 0.17–0.77; *P =* 0.0072), but not in the placebo‐chemo arm (Figure 3A–C).

In the metastatic setting, ICIs blocking the PD‐1/PD‐L1 axis are currently approved in combination with chemotherapy only for PD‐L1^positive^ mTNBC. However, in the ALICE study, the benefit of atezolizumab was independent of PD‐L1 expression. Evaluation of PD‐L1 expression was performed as previously described [15]. Among patients with baseline fecal samples, PD‐L1 status was known for 58 of 59 patients and 50% (29/58) of these patients had PD‐L1‐positive biopsies. We found no association between Faith's PD and PD‐L1 status (Figure S5).

### 3.3 Prognostic value of alpha diversity after controlling for other prognostic factors

To control for other factors influencing PFS, we performed univariate and bivariate Cox proportional hazards analyses. In the univariate model, high Faith's PD (*P =* 0.011), older age (*P =* 0.003) and less than three metastatic sites (*P =* 0.014) were significantly associated with improved PFS (Table S3). The presence of liver metastases gave a trend toward inferior PFS (*P =* 0.054). In the bivariate model, we adjusted Faith's PD for each of these factors and found that the HRs remained largely unchanged. Faith's PD remained statistically significant after controlling for age (HR 0.55, *P* = 0.041) or the number of metastatic sites (HR 0.47, *P* = 0.009), and borderline significant after adjusting for liver metastases (HR 0.54, *P* = 0.051) (Table S4). To investigate if the prognostic value of Faith's PD was dependent on the cut‐off, we also performed a Cox analysis with Faith's PD as a continuous variable. The univariate analysis showed that Faith's PD as a continuous variable was also significantly associated with PFS (HR 0.45, *P* = 0.016; Table S4).

### 3.4 High alpha diversity is associated with response to chemo‐immunotherapy

**FIGURE 4:**
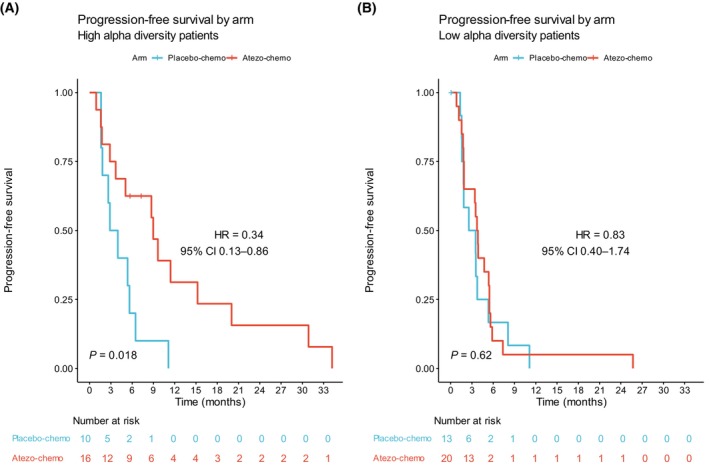
Kaplan–Meier plots of progression‐free survival by treatment arm in low and high diversity patients. Kaplan–Meier plots of progression‐free survival in the atezo‐chemo arm compared to the placebo‐chemo arm in (A) patients with high Faith's phylogenetic diversity and (B) patients with low Faith's phylogenetic diversity. Patients were classified into low and high diversity groups based on the optimized cut‐off of Faith's phylogenetic diversity. Hazard ratios and 95% confidence intervals were calculated using the Cox proportional hazards model. *P* values were calculated by the log‐rank method.

Furthermore, we evaluated if Faith's PD could serve as a predictive biomarker of benefit from atezo‐chemo. Using the optimized cut‐off, we found that patients with high Faith's PD had a clear PFS benefit from atezo‐chemo (HR 0.34, 95% CI 0.13–0.86; *P* = 0.018; Figure 4A). By contrast, for patients with low Faith's PD, we observed no significant difference in PFS between the treatment arms (HR 0.83, 95% CI 0.40–1.74; *P* = 0.62; Figure 4B). The findings thus indicate that high gut microbiota diversity by Faith's PD was predictive of benefit from the addition of atezolizumab to chemotherapy in ALICE.

### 3.5 Association of baseline gut microbiota composition and clinical benefit

The bacterial abundance at genus level for each patient is given and available in Table S5.

**FIGURE 5:**
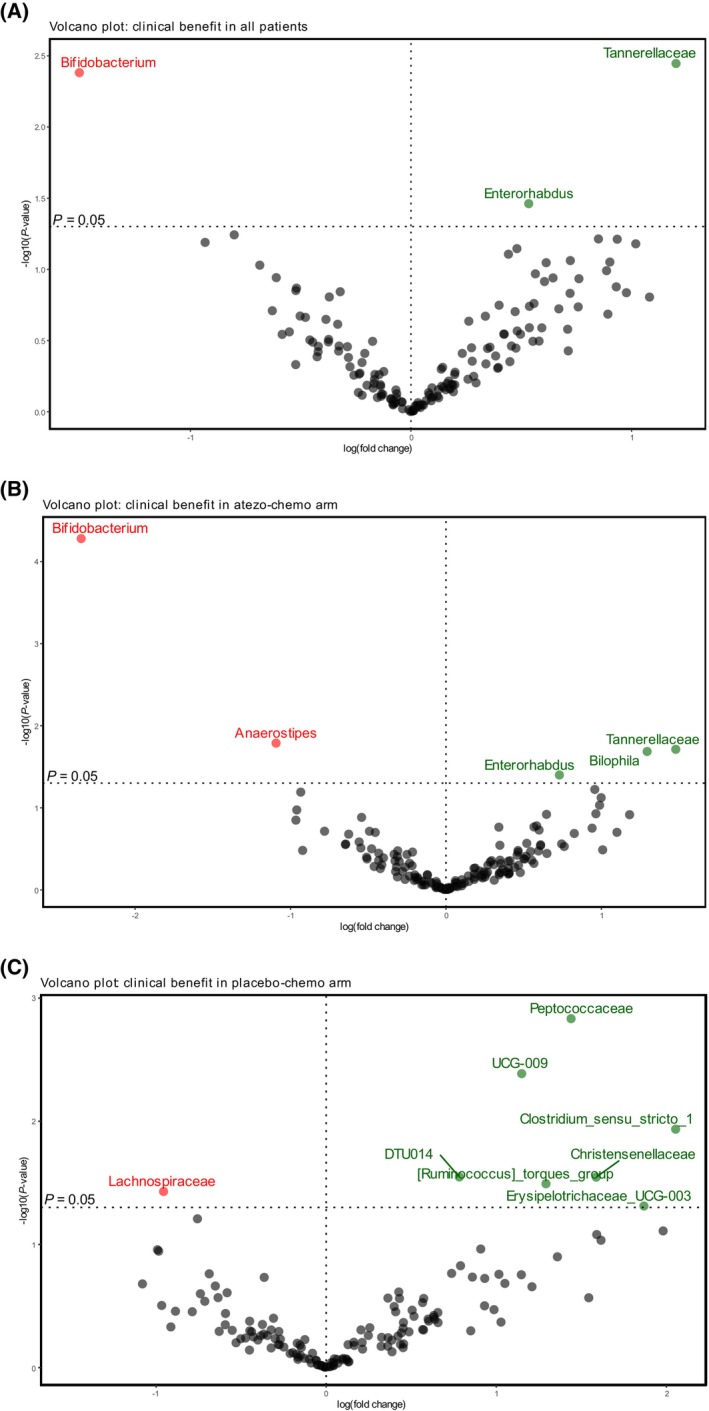
Differential abundance analysis (using ANCOM‐BC2) according to clinical benefit. Volcano plot on (A) all patients, (B) the atezo‐chemo and (C) the placebo‐chemo arm. The plot visualizes differentially abundant bacterial genera in patients with and without clinical benefit. X‐axis represents effect size and y‐axis represents *P* values. Taxa with *P* value <0.05 are presented above the horizontal dashed line. Taxa enriched in patients with clinical benefit are colored in green and taxa enriched in patients without clinical benefit are colored in red. The vertical dashed line illustrates log fold change of zero.

To explore potential associations between bacterial taxa and clinical outcome, we used the categorical variable clinical benefit, as defined in the ALICE protocol. ANCOM‐BC2 was employed to identify taxa differentially enriched in patients with and without clinical benefit. In all patients, we found that *Tannerellaceae* and *Enterorhabdus* were enriched in patients with clinical benefit. *Bifidobacterium* was enriched in patients without clinical benefit (Figure 5A). Next, we analyzed the two treatment arms separately. In the atezo‐chemo arm *Tannerellaceae*, *Bilophila* and *Enterorhabdus* were enriched in patients with clinical benefit. We found that *Bifidobacterium and Anaerostipes* were significantly overrepresented in patients without clinical benefit in the atezo‐chemo arm (Figure 5B), this was not observed in the placebo‐chemo arm (Figure 5C). The dominance of *Bifidobacterium* in patients without clinical benefit in the atezo‐chemo arm was the only statistically significant finding after correction for multiple testing in ANCOM‐BC2 (*q* = 0.008). Among all patients, we found that patients with high relative abundance of *Bifidobacterium* had significantly decreased Faith's PD compared to patients with low abundance of *Bifidobacterium* (Figure S6).

The negative association between high abundance of *Bifidobacterium* and clinical benefit is in contrast to studies in other tumor types where *Bifidobacterium* has generally been associated with response to checkpoint inhibitors [33]. As technical validation, we performed a second 16S rRNA gene sequencing analysis of the baseline samples from the atezo‐chemo group at another laboratory. The relative abundance of *Bifidobacterium* was strongly correlated in the two sequencing experiments (Kendall's tau 0.83, *P* = 1.3e‐12).

### 3.6 Association of baseline gut microbiota and irAEs

**FIGURE 6:**
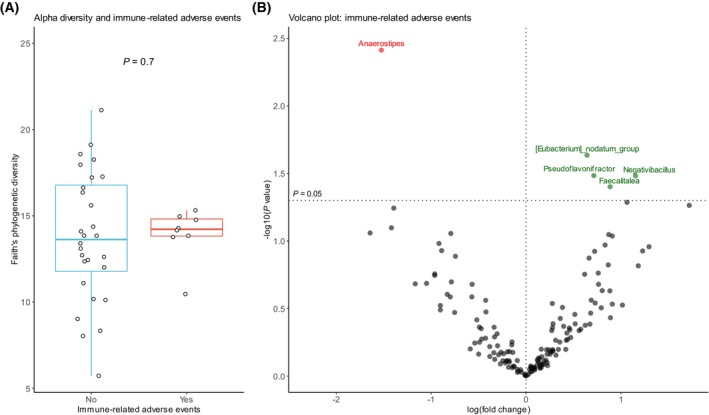
Immune‐related adverse events. (A) Alpha diversity by Faith's phylogenetic diversity in patients with and without immune‐related adverse events (irAEs) of any grade. *P* value calculated by Wilcoxon rank sum test. The box plot extends from the first to the third quartile. The middlle line represents the median and the whiskers to the most extreme point within 1.5 × IQR. (B) Volcano plot based on ANCOM‐BC2. The plot visualizes differentially abundant bacterial genera in patients with and without irAEs (any grade) in the atezo‐chemo arm. X‐axis represents effect size and y‐axis represents *P* values. Taxa with *P* value <0.05 are presented above the horizontal dashed line. Taxa enriched in patients with irAEs are colored in green and taxa enriched in patients without irAEs are colored in red. The vertical dashed line illustrates log fold change of zero. No taxa were statistically significant after correction for multiple testing in ANCOM‐BC2.

The intestinal microbiota has been associated with irAE development in melanoma [3, 34]. We compared baseline microbiota composition in patients with and without irAEs in the atezo‐chemo arm. IrAEs of any grade were documented in 22% of the atezo‐chemo arm. No association between Faith's PD and irAEs was observed (*P* = 0.70, Figure 6A). We used ANCOM‐BC2 to identify taxa associated with irAEs. *Eubacterium nodatum* group, *Pseudoflavonifractor*, *Negativibacillus* and *Faecalitalea* were overrepresented in patients with irAEs. *Anaerostipes* was enriched in patients without irAEs (Figure 6B).

### 3.7 Assessment of the gut microbiota during treatment

**FIGURE 7:**
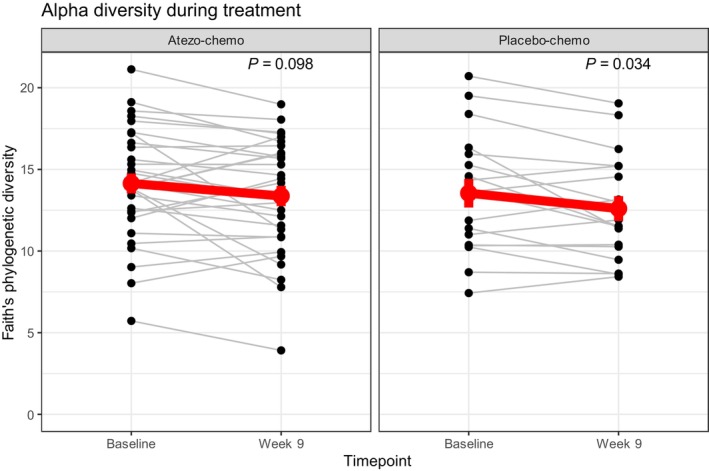
Alpha diversity during treatment in the two arms. Paired samples from patients that provided samples at baseline and week 9 demonstrated that alpha diversity decreased during treatment, both in the atezo‐chemo arm (left) and in the placebo‐chemo arm (right). *P* values were calculated using Wilcoxon signed‐rank test. The red point represents the mean alpha diveristy at each timepoint and the errorbars represent the standard error of the mean. The red line represents the change of the mean alpha diversity value from baseline to week 9.

We further investigated changes in gut microbiota during treatment by comparing microbiota diversity and composition at baseline to fecal samples collected after 8 weeks of treatment (week 9, *n* = 49). Paired analysis demonstrated that Faith's PD was significantly reduced during treatment (*P* = 0.0069). This applied to both arms, but the change only reached statistical significance in the placebo‐chemo arm (Figure 7).

When assessing changes in the gut microbiota composition during treatment, we observed that the mean relative abundance of *Bifidobacterium* significantly increased after 8 weeks of treatment in the atezo‐chemo arm, but did not differ significantly in the placebo‐chemo arm (Figure S7a,b). However, when analyzing patients with and without clinical benefit separately in the atezo‐chemo arm, we found that *Bifidobacterium* only increased significantly in patients with clinical benefit. For patients without clinical benefit in the atezo‐chemo arm, the relative abundance of *Bifidobacterium* was stable during treatment (Figure S8).

## 4 Discussion

The ALICE trial demonstrated clinical benefit in mTNBC from adding atezolizumab to immunomodulatory chemotherapy, regardless of PD‐L1 tumor status [15]. The clinical responses were highly variable between the patients, highlighting the need for biomarkers and for a better understanding of mechanisms for effect and resistance to immunotherapy. In the present study, we analyzed the dynamics, and the prognostic and predictive value of the gut microbiota for mTNBC patients treated in the ALICE trial. To our knowledge, this is the first study to assess the gut microbiota in mTNBC receiving chemo‐immunotherapy. We show that a high baseline alpha diversity by Faith's PD was significantly associated with prolonged PFS in the ALICE trial. Interestingly, this observation only applied to patients receiving atezolizumab, not to those only receiving chemotherapy. Bivariate analyses supported an independent prognostic value of Faith's PD, after correction for other factors. Moreover, we found that the PFS benefit from addition of atezolizumab to chemotherapy appeared to be confined to patients with a high Faith's PD. Our data thus suggest that high alpha diversity, as measured by Faith's PD, is a candidate predictive biomarker for response to this form of chemo‐immunotherapy.

High alpha diversity is usually linked to a healthy gut microbiota [35]. In immuno‐oncology, one study found an association between PFS and alpha diversity in melanoma patients treated with PD‐1 blockade [36]. However, larger studies were not able to reproduce this observation [2, 3]. In patients with non‐small cell lung cancer and renal cell carcinoma treated with PD‐1 inhibitors, studies have reported an association between high alpha diversity and a favorable outcome, when excluding patients treated with antibiotics [37, 38]. In breast cancer, high baseline gut microbiota alpha diversity has been associated with response to both neoadjuvant HER2 blockade and neoadjuvant chemotherapy [8, 12].

The lack of standardized readouts for diversity and other features is a major challenge within the field of microbiota research. Different studies have employed different sequencing platforms, clinical outcome measures and indices of microbiota diversity. This makes cross‐study comparisons on alpha diversity and its association with clinical outcome difficult. In our study, we did not observe a statistically significant association between observed ASVs and PFS. This observation illustrates that these indexes, though both measuring alpha diversity, are based on different algorithms. Observed ASVs only provide information on richness, whereas Faith's PD is a richness metric incorporating phylogenetic similarity between taxa. From the literature, it is not known which index may be more predictive of immunotherapy responses. The findings in our study underline the need for validating any findings using the same algorithms. Furthermore, it may be of interest to re‐analyze data from previous studies with alternative algorithms for alpha diversity.

The apparent predictive value of Faith's PD in our dataset requires validation in an independent cohort. However, the concept that high alpha diversity is beneficial for immune responses is in line with current knowledge within the field, as mentioned above, and attempts to identify an alpha diversity index with predictive value for immunotherapy would be warranted. If Faith's PD were to be used for biomarker purposes, the cut‐off would need to be further investigated. The optimized cut‐off identified in our explorative study suggests that an appropriate cut‐off may be above the median in mTNBC patients, but this would have to be established in independent cohorts. Further, the use of Faith's PD as a biomarker in clinical practice would require the development of a single‐sample predictor, as well as standardized procedures for feces collection, storage and analysis.

We further investigated taxa associated with benefit from or resistance to therapy. The results revealed that *Tannerellaceae* and *Enterorhabdus* were enriched in patients with clinical benefit, regardless of treatment arm. These observations were only significant if correction for multiple testing was not applied, and may be incidental, but provide a basis for further studies. One surprising finding in our study was that *Bifidobacterium* at baseline was overrepresented in patients without clinical benefit in the atezo‐chemo arm. This finding remained significant after correction for multiple testing. The genus *Bifidobacterium* has generally been associated with favorable responses to ICIs [33], although this has not yet been evaluated in breast cancer. Further, different bacterial taxa have been associated with ICI outcome across different studies. This was emphasized in a recent meta‐ and mega‐analysis that investigated gut microbiota signatures associated with response and resistance to ICIs across different cancer types [5]. This study analyzed stool samples from patients with non‐small cell lung cancer, melanoma and renal cell carcinoma in 12 cohorts from eight published studies. The authors observed limited overlap across different studies when examining taxonomic biomarkers associated with response or resistance. We validated our observations on *Bifidobacterium* in a second 16S rRNA gene sequencing experiment, which confirmed the finding. Furthermore, longitudinal analysis demonstrated that *Bifidobacterium* significantly increased during treatment in the atezo‐chemo arm, but only in patients with clinical benefit from treatment. In our data set, clinical benefit was thus associated with lower *Bifidobacterium* levels at baseline, but also with an increase in *Bifidobacterium* during treatment. These observations should be interpreted with caution. However, the findings suggest that the interaction between microbiota and clinical outcome is complex and that the role of specific bacterial taxa may depend on the time point of sampling, as well as the patient population, treatment and other factors. This issue is worth recalling when considering microbiota interventions, which should be investigated and validated in precisely defined patient cohorts.

We observed a reduction in alpha diversity during treatment in both trial arms. This is in line with other studies reporting that chemotherapy leads to a reduction in alpha diversity (reviewed in [39]). Based on the associations between diversity and treatment effect, this could raise a concern that the effect of therapy would decrease over time. It is conceivable that the effect of therapy on microbiota could contribute to therapy resistance, and even to the common observation that patients respond more poorly in later treatment lines. The causes of these clinical challenges are surely complex, but further studies would be of interest, as microbiota diversity may be possible to modulate through interventions.

Another potential factor which could influence diversity and treatment effect is antibiotics. Antimicrobial therapy has been associated with worse survival in TNBC treated with curative intent [40]. Only five patients received antibiotics within the period recorded in our study (last 30 days before enrolment, or between baseline and week 9). Due to this low sample size, we did not analyze the effect of antibiotic exposure. This highlights important limitations in our study. While the proportion of patients providing fecal samples was high (87%), the relatively small sample size does represent a limitation, as do limited data on lifestyle factors such as diet, that presumably contribute to microbiota composition and diversity. In particular, the sample sizes for analyses of each study arm separately were small. Another important limitation is the fact that the study is explorative. The findings should therefore be interpreted with caution and in context with data from other studies, and further investigated in other cohorts.

## 5 Conclusions

In conclusion, our study suggests that a high gut microbiota alpha diversity by Faith's PD was associated with improved clinical outcome in mTNBC patients receiving chemo‐immunotherapy and with benefit from atezolizumab. These findings require validation in independent cohorts, but may be of clinical importance, as the effect of immunotherapy is highly variable among mTNBC patients. We also identify taxa appearing to be associated with benefit or resistance to chemotherapy alone and chemo‐immunotherapy. Further studies exploring Faith's PD and other candidate biomarkers for the effect of immunotherapy in mTNBC are warranted. Moreover, improved understanding of the role of microbiota in tuning anti‐cancer responses may inform targeted interventions aimed at creating a beneficial microbiota profile.

## Conflict of interest

J.A.K. has the last five years received research support from Bristol Myers Squibb, F. Hoffmann‐La Roche, NanoString and NEC OncoImmunity and has previously received advisory board/lecture honoraria from pharmaceutical companies, including Roche. JRH has received grants from Biogen and lecture honoraria from Amgen, Roche and Novartis. The other authors declare no conflicts of interest.

## Author contributions

JAK was the coordinating investigator and medical monitor of the ALICE trial and responsible for study design and acquisition of funding and approvals. AHR and NKA were investigators at Oslo University Hospital and medical monitors for the other sites, and contributed to patient recruitment and data collection. The present study was designed by JAK, JRH and AU. CB was responsible for stool sample processing and 16S rRNA gene sequencing. AU, AHR and KH analyzed data. JAK, AU, AHR, NKA, KH, JRH, BN and OCL interpreted data. The manuscript was written by JAK and AU, with contributions from all authors. All authors read and approved the manuscript.

## Supporting information


**Fig. S1.** Progression‐free survival for patients that provided fecal samples at baseline in ALICE.
**Fig. S2.** Progression‐free survival by baseline observed ASVs.
**Fig. S3.** Alpha diversity in patients with PFS >6 months and patients with PFS ≤6 months.
**Fig. S4.** Optimal cut‐off for Faith's phylogenetic diversity.
**Fig. S5.** Alpha diversity and PD‐L1 status.
**Fig. S6.** Alpha diversity and relative abundance of *Bifidobacterium*.
**Fig. S7.** Taxa‐specific changes during treatment in the treatment arms.
**Fig. S8.** Bifidobacterium dynamics during treatment stratified according to clinical benefit.

**Table S1.** Baseline characteristics.

**Table S2.** Per‐sample characteristics.

**Table S3.** Univariate Cox regression model for progression‐free survival.

**Table S4.** Multivariate Cox regression model for progression‐free survival.

**Table S5.** Non rarefied feature table with bacterial abundance for each sample.

## Data Availability

Data are not deposited in a public repository due to data privacy regulations in Norway and our institution. However, data are available upon request, if the aims of the planned analyses are covered by the written informed consent signed by the participants, pending an amendment to the ethical approvals and a material & data transfer agreement between the institutions. The transfer of data or materials will require approval from the Data Privacy Officer and Institutional Review Board at OUH, and from the Regional Committee for Medical and Health Research Ethics South‐East Norway and the Research Ethics Committee in Denmark. Any shared data will be de‐identified. Requests should be made to the corresponding author (jonky@ous-hf.no).
