# Supplementary material for: Gut microbiota diversity is prognostic and associated with benefit from chemo‐immunotherapy in metastatic triple‐negative breast cancer
Source: Mol Oncol. 2024 Nov 15;19(4):1229–43. doi: 10.1002/1878-0261.13760 (PMC11977656; doi:10.1002/1878-0261.13760)
Supplement: Supplementary file 1 — Fig. S1. Progression‐free survival for patients that provided fecal samples at baseline in ALICE. Fig. S2. Progression‐free survival by baseline observed ASVs. Fig. S3. Alpha diversity in patients with PFS >6 months and patients with PFS ≤6 months. Fig. S4. Optimal cut‐off for Faith's phylogenetic diversity. Fig. S5. Alpha diversity and PD‐L1 status. Fig. S6. Alpha diversity and relative abundance of Bifidobacterium. Fig. S7. Taxa‐specific changes during treatment in the treatment arms. Fig. S8. Bifidobacterium dynamics during treatment stratified according to clinical benefit. [file MOL2-19-1229-s005.pdf]

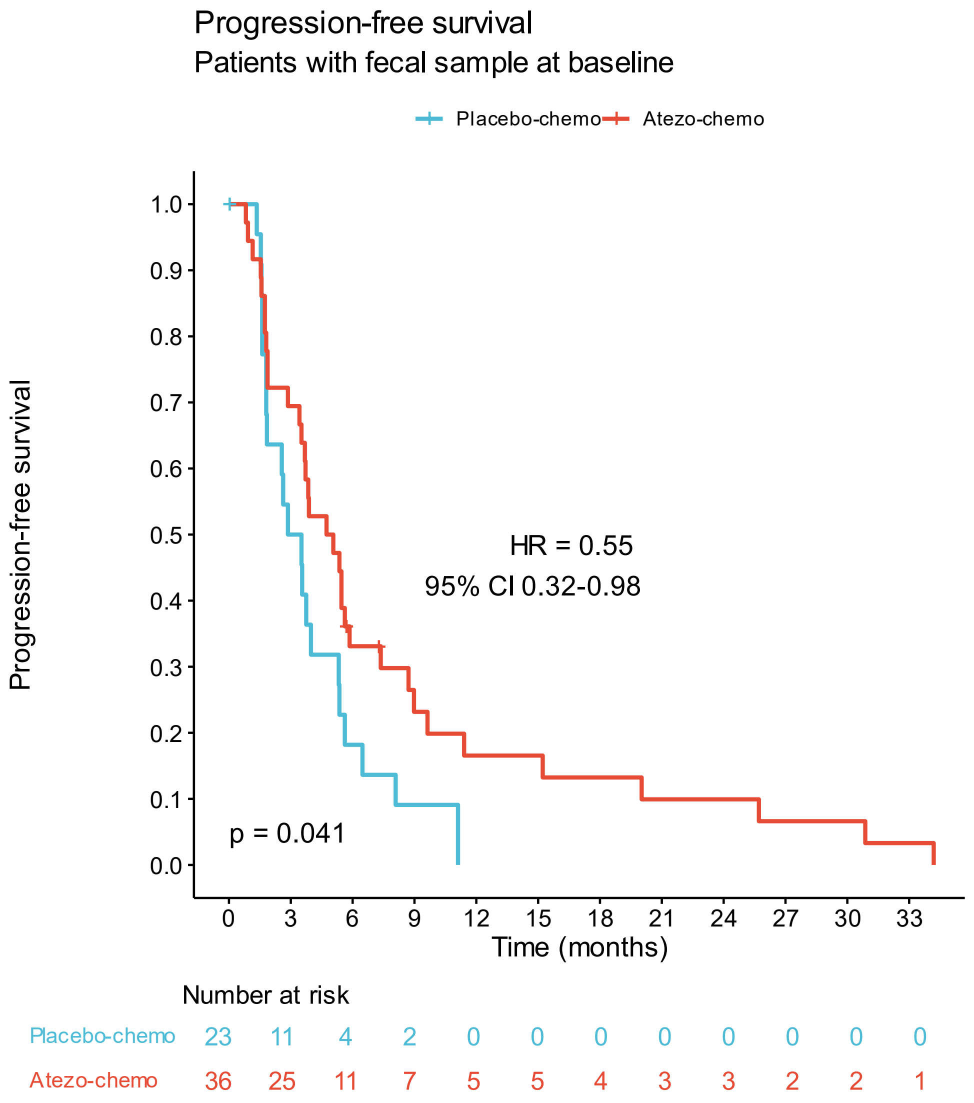

Figure S1. Progression-free survival for patients that provided fecal sample at baseline in ALICE

Hazard ratios and 95% confidence intervals were calculated using the Cox proportional hazards model. *P* value calculated by the log-rank method.

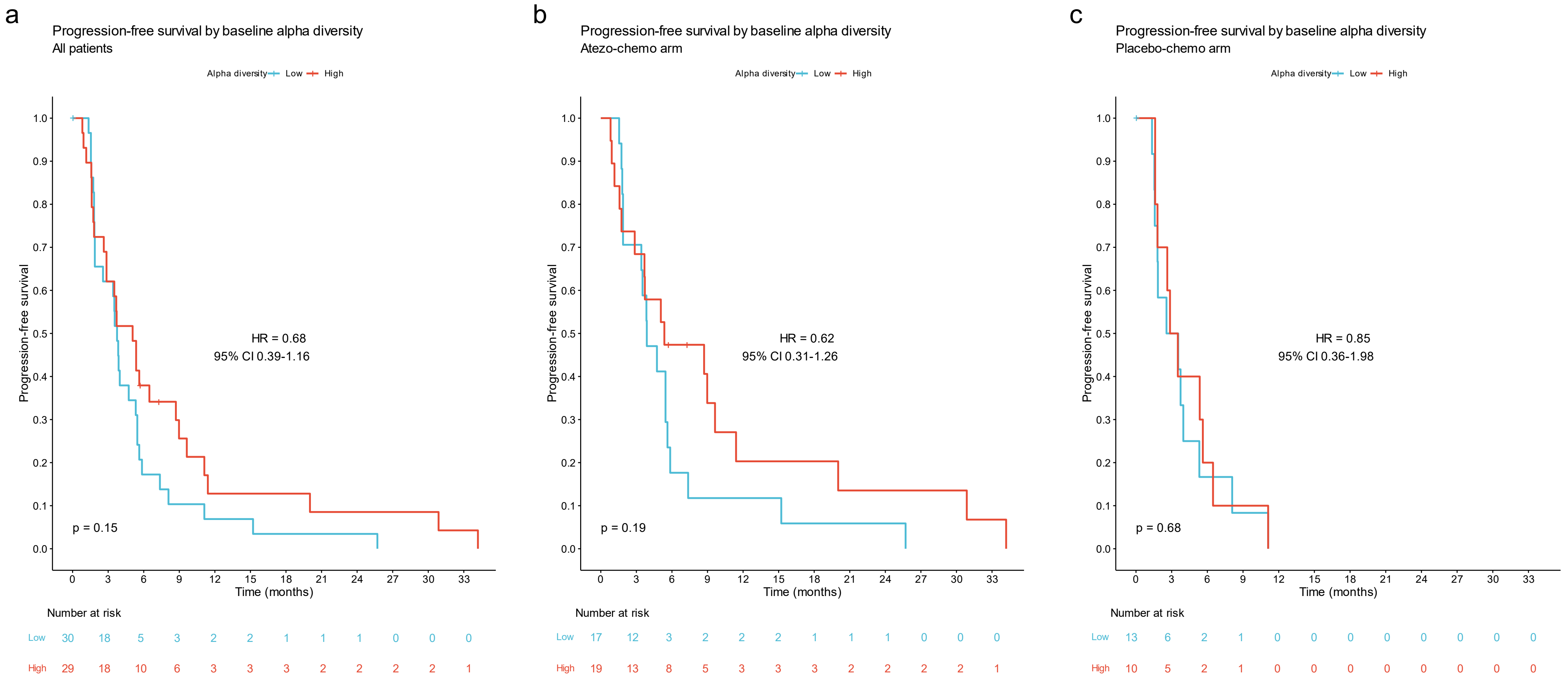

Figure S2. Progression-free survival by baseline observed ASVs

Kaplan-Meier plot in (a) all patients, (b) the atezo-chemo and (c) the placebo-chemo arm.
Patients were classified into low and high diversity groups based on the median score of observed ASVs. Hazard ratios and 95% confidence intervals were calculated using the Cox proportional hazards model. *P* values were calculated by the log-rank method.

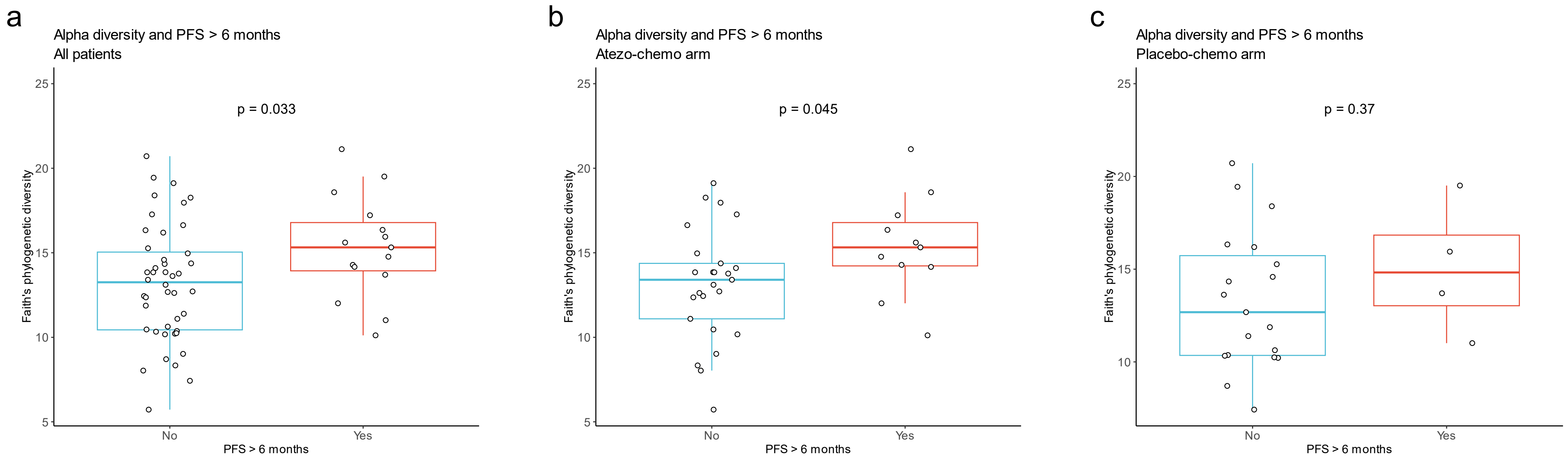

Figure S3. Alpha diversity in patients with PFS > 6 months and patients with PFS ≤ 6 months

Faith's phylogenetic diversity in patients with PFS > 6 months and patients with PFS ≤ 6 months, in (a) all patients, (b) the atezo-chemo and (c) the placebo-chemo arm. *P* values calculated by Wilcoxon rank sum test. The box plot extends from the first to the third quartile. The middlle line represents the median and the whiskers to the most extreme point within 1.5 x IQR. Each dot represents a sample.

a

Cutoff (percentile)	*P*-value
56	0.007
63	0.012
54	0.020
68	0.023
58	0.024
59	0.031
53	0.037
66	0.039
71	0.040

b

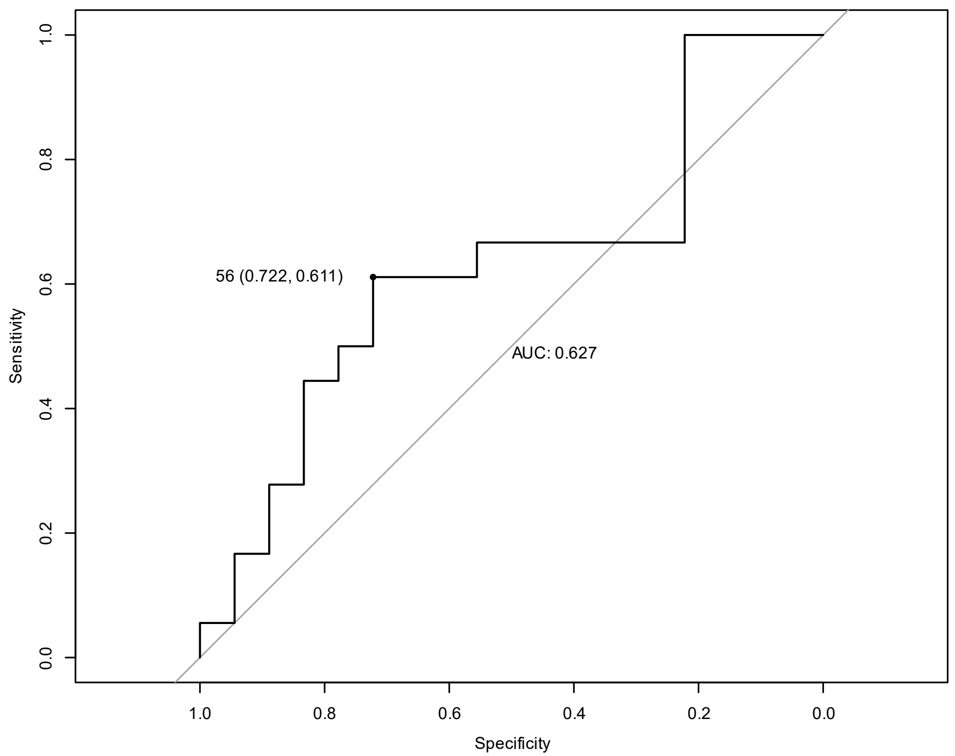

Figure S4. Optimal cut-off for Faith's phylogenetic diversity

(a) The optimal cutoff value for Faith's PD was based on confidence level and group size (minimum 20% in each group). A table was constructed using the log-rank method for progression-free survival testing all cut-offs of Faith's PD in the atezo-chemo arm. The table in (a) shows cut-offs (percentiles) with *P* value < 0.05 with minimum 20 % of patients in each group. The cut-off with lowest *P* value was selected. Percentiles are calculated based on the total study population.

(b) ROC curve with progression-free survival (dichotomized by the median value) as a response variable and Faith's PD as a predictor variable. The optimal cutoff value of Faith's PD in the atezo-chemo arm (corresponding to the 56th percentile in the total study population) is shown.

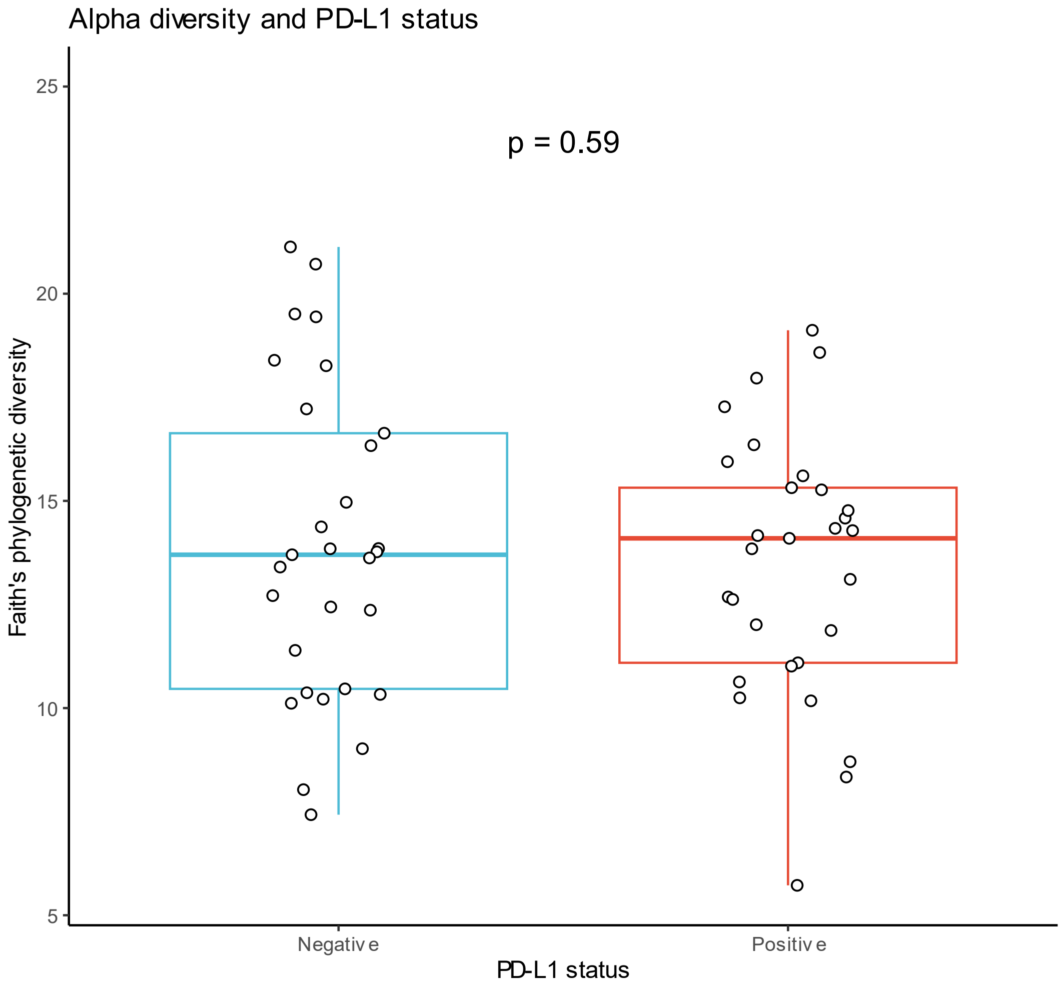

Figure S5. Alpha diversity and PD-L1 status

Faith's PD in patients with PD-L1 positive and negative tumores. PD-L1 status known for 58 of the 59 patients with fecal sample at baseline. PD-L1 scoring performed on archival pre-study biopsies. *P* value calculated by two-sample t-test. The box plot extends from the first to the third quartile. The middlle line represents the median and the whiskers to the most extreme point within 1.5 x IQR. Each dot represents a sample.

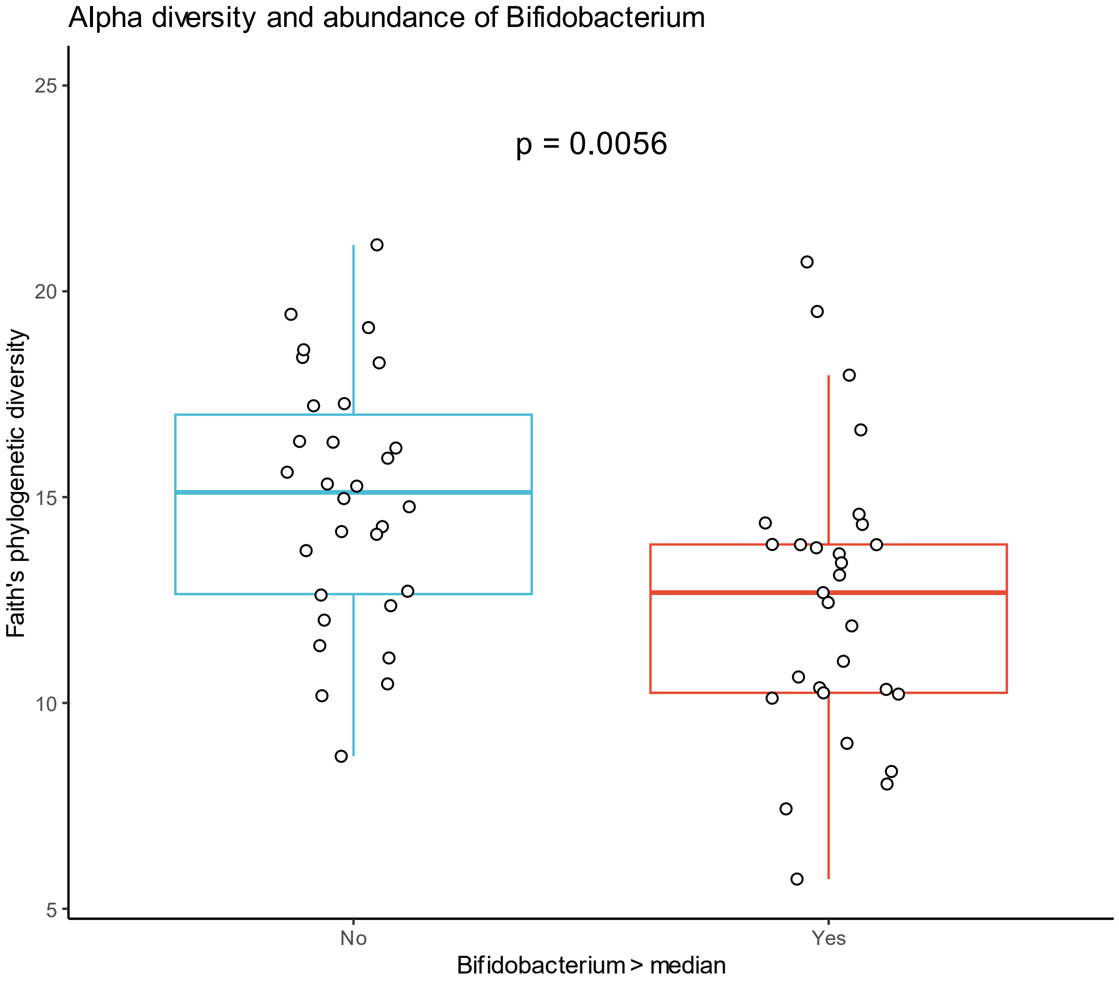

Figure S6. Alpha diversity and relative abundance of *Bifidobacterium*

High relative abundance (>median) of *Bifidobacterium* was associated with reduced alpha diversity by Faith's PD in all patients. *P* value calculated by two-sample t-test. The box plot extends from the first to the third quartile. The middlle line represents the median and the whiskers to the most extreme point within 1.5 x IQR. Each dot represents a sample.

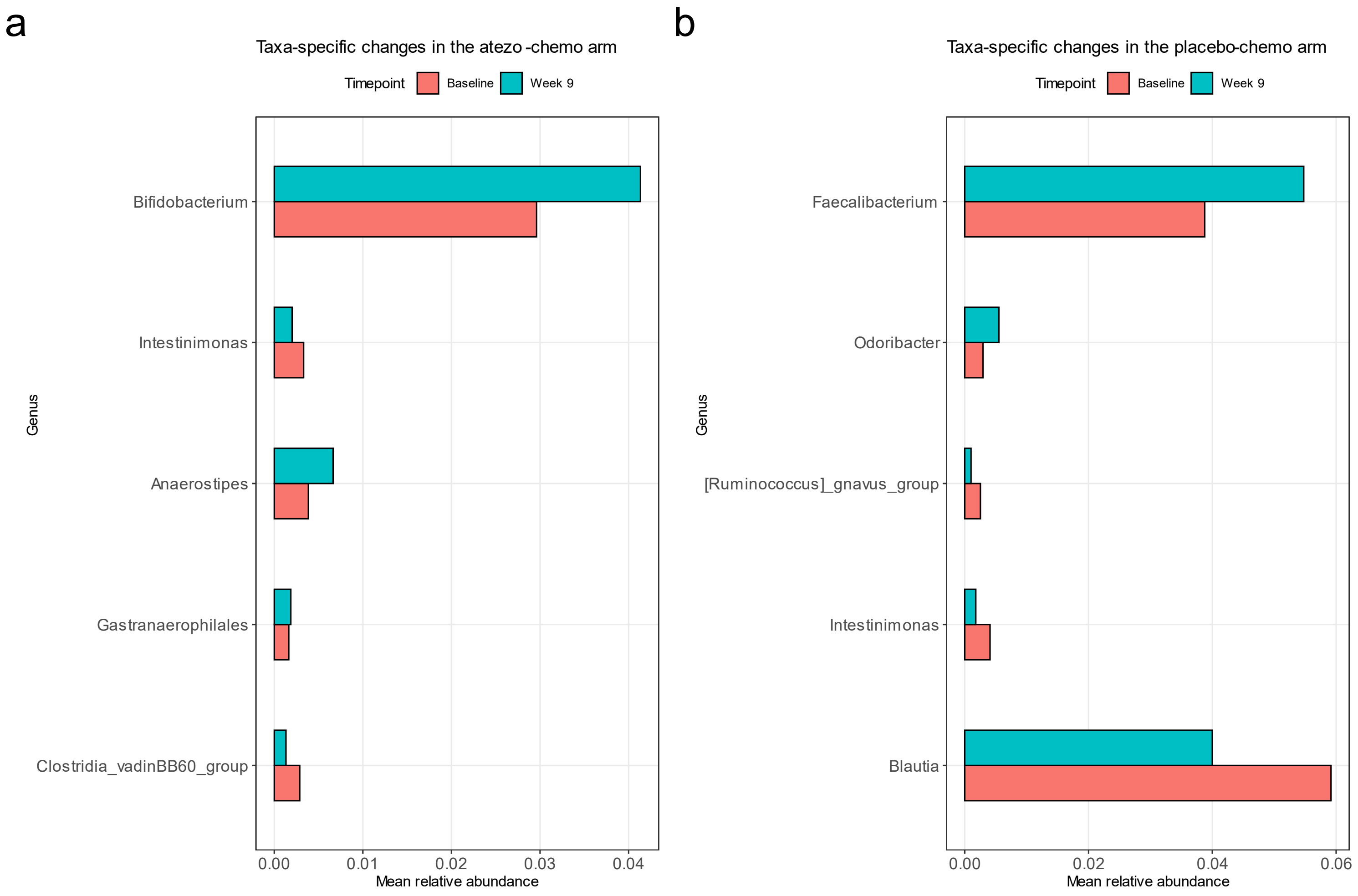

Figure S7. Taxa-specific changes during treatment in the treatment arms

Plot showing taxa with signifcant change (*P*<0.05) from baseline to week 9 in the atezo-chemo arm (a) and placebo-chemo arm (b) from paired samples. X-axis represents mean relative abdundance and y-axis represents taxa. *P* values were calculated using Wilcoxon signed-rank test.

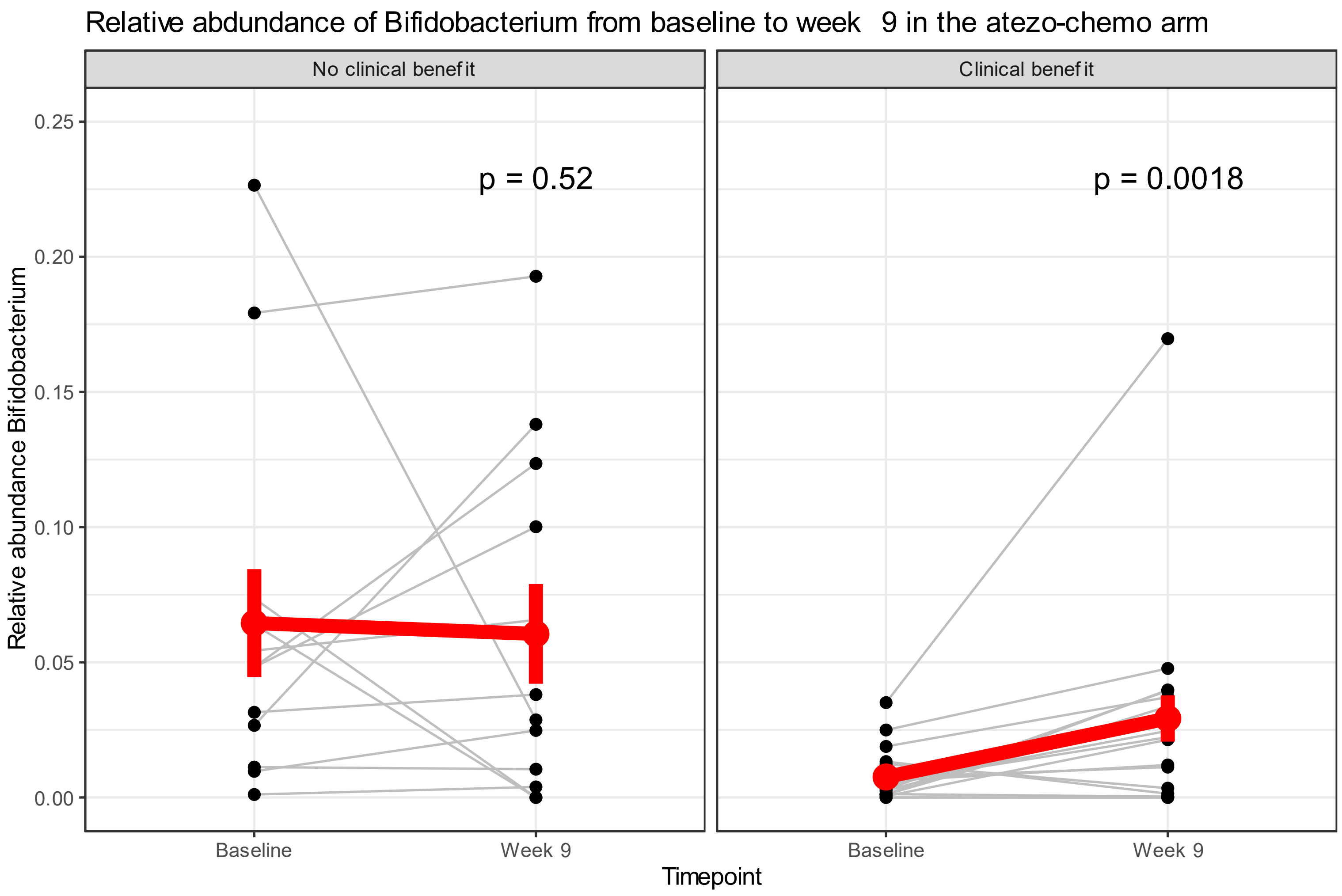

Figure S8. Bifidobacterium dynamics during treatment stratified according to clinical benefit

Paired samples from patients in the atezo-chemo arm that provided samples at baseline and week 9. The relative abundance of *Bifidobacterium* increased in patients with clincal benefit. *P* values were calculated using Wilcoxon signed-rank test. The red point represents the mean alpha diveristy at each timepoint and the errorbars represent the standard error of the mean. The red line represents the change of the mean alpha diversity value from baseline to week 9.
