# Supplementary material for: Gut microbiota diversity is prognostic and associated with benefit from chemo‐immunotherapy in metastatic triple‐negative breast cancer
Source: Mol Oncol. 2024 Nov 15;19(4):1229–43. doi: 10.1002/1878-0261.13760 (PMC11977656; doi:10.1002/1878-0261.13760)
Supplement: Supplementary file 2 — Table S1. Baseline characteristics. [file MOL2-19-1229-s003.pdf]

	Atezolizumab + Chemo (N=36)	Placebo + Chemo (N=23)
**Age (years)**		
Median [range]	58.5 [31.0, 77.0]	53.0 [31.0, 74.0]
**Gender**		
Female	35 (97.2%)	23 (100%)
Male	1 (2.8%)	0 (0%)
**ECOG**		
0	25 (69.4%)	18 (78.3%)
1	11 (30.6%)	5 (21.7%)
**De novo metastatic disease**		
Yes	9 (25.0%)	6 (26.1%)
No	27 (75.0%)	17 (73.9%)
**Line of metastatic treatment**		
1st	22 (61.1%)	12 (52.2%)
2nd	14 (38.9%)	11 (47.8%)
**Liver metastases**		
Yes	12 (33.3%)	9 (39.1%)
No	24 (66.7%)	14 (60.9%)
**Lung metastases**		
Yes	17 (47.2%)	9 (39.1%)
No	19 (52.8%)	14 (60.9%)
**PD-L1 status**		
Positive	19 (52.8%)	10 (43.5%)
Negative	17 (47.2%)	12 (52.2%)
Missing	0 (0%)	1 (4.3%)
**Previous adjuvant chemotherapy**		
Yes	24 (66.7%)	17 (73.9%)
No	12 (33.3%)	6 (26.1%)
**Number of metastatic sites**		
≤2	25 (69.4%)	12 (52.2%)
>2	11 (30.6%)	11 (47.8%)

Table S1. Baseline characteristics

Abbreviations: N, number of patients; ECOG, Eastern Cooperative Oncology Group; PD-L1, programmed death-ligand 1.
