# Supplementary material for: Gut microbiota diversity is prognostic and associated with benefit from chemo‐immunotherapy in metastatic triple‐negative breast cancer
Source: Mol Oncol. 2024 Nov 15;19(4):1229–43. doi: 10.1002/1878-0261.13760 (PMC11977656; doi:10.1002/1878-0261.13760)
Supplement: Supplementary file 3 — Table S2. Per‐sample characteristics. [file MOL2-19-1229-s006.pdf]

Table S2

PatientID[1]	Timepoint	time.pfs[2]	event.pfs	PD-L1	Faith PD[3]	High Faith PD[4]	Observed ASVs[5]	High observed ASVs[6]	Treatment arm	Immune-related adverse event[7]	Clinical benefit
p001	Week 9	4.73	Yes	Negative	9.94		119		Atezo-chemo	No	No
p001	Baseline	4.73	Yes	Negative	9.02	No	104	No	Atezo-chemo	No	No
p002	Baseline	0.82	Yes	Positive	14.10	Yes	186	Yes	Atezo-chemo	No	No
p002	Week 9	0.82	Yes	Positive	12.50		161		Atezo-chemo	No	No
p003	Baseline	15.22	Yes	Positive	14.16	Yes	169	No	Atezo-chemo	Yes	Yes
p003	Week 9	15.22	Yes	Positive	16.99		233		Atezo-chemo	Yes	Yes
p004	Week 9	1.58	Yes	Negative	10.39		146		Placebo-chemo	No	No
p004	Baseline	1.58	Yes	Negative	10.33	No	128	No	Placebo-chemo	No	No
p005	Week 9	34.19	Yes	Negative	11.33		153		Atezo-chemo	No	Yes
p005	Baseline	34.19	Yes	Negative	17.22	Yes	227	Yes	Atezo-chemo	No	Yes
p006	Week 9	2.56	Yes	Negative	9.47		119		Placebo-chemo	No	No
p006	Baseline	2.56	Yes	Negative	11.39	No	137	No	Placebo-chemo	No	No
p007	Week 9	5.36	Yes	Positive	12.99		148		Placebo-chemo	Yes	Yes
p007	Baseline	5.36	Yes	Positive	15.27	Yes	186	Yes	Placebo-chemo	Yes	Yes
p008	Baseline	30.87	Yes	Negative	21.13	Yes	255	Yes	Atezo-chemo	No	Yes
p008	Week 9	30.87	Yes	Negative	18.99		241		Atezo-chemo	No	Yes
p009	Baseline	5.85	Yes	Positive	11.09	No	123	No	Atezo-chemo	No	Yes
p009	Week 9	5.85	Yes	Positive	10.85		101		Atezo-chemo	No	Yes
p010	Week 9	11.11	Yes	Negative	11.52		145		Placebo-chemo	No	Yes
p010	Baseline	11.11	Yes	Negative	13.70	No	182	No	Placebo-chemo	No	Yes
p011	Week 9	20.02	Yes	Positive	13.20		162		Atezo-chemo	Yes	Yes
p011	Baseline	20.02	Yes	Positive	14.77	Yes	184	Yes	Atezo-chemo	Yes	Yes
p012	Week 9	3.98	Yes	Positive	11.53		134		Placebo-chemo	No	No
p012	Baseline	3.98	Yes	Positive	14.58	Yes	177	No	Placebo-chemo	No	No
p013	Baseline	1.55	Yes	Positive	8.34	No	103	No	Atezo-chemo	No	No
p014	Week 9	7.27	No	Positive	18.05		253		Atezo-chemo	No	Yes
p014	Baseline	7.27	No	Positive	18.58	Yes	257	Yes	Atezo-chemo	No	Yes
p015	Baseline	2.86	Yes	Negative	18.39	Yes	251	Yes	Placebo-chemo	No	No
p015	Week 9	2.86	Yes	Negative	16.25		201		Placebo-chemo	No	No
p016	Week 9	1.74	Yes	Negative	15.68		199		Atezo-chemo	No	No
p016	Baseline	1.74	Yes	Negative	16.63	Yes	215	Yes	Atezo-chemo	No	No
p017	Baseline	0.92	Yes	Negative	14.37	Yes	189	Yes	Atezo-chemo	No	No
p018	Week 9	5.46	Yes	Negative	14.18		194		Atezo-chemo	No	Yes
p018	Baseline	5.46	Yes	Negative	12.36	No	168	No	Atezo-chemo	No	Yes
p019	Baseline	3.75	Yes	Positive	8.70	No	116	No	Placebo-chemo	Yes	No
p019	Week 9	3.75	Yes	Positive	8.62		115		Placebo-chemo	Yes	No
p020	Baseline	5.33	Yes	Negative	10.37	No	135	No	Placebo-chemo	No	Yes
p020	Week 9	5.33	Yes	Negative	10.27		122		Placebo-chemo	No	Yes
p021	Week 9	2.86	Yes	Positive	16.71		225		Atezo-chemo	No	No
p021	Baseline	2.86	Yes	Positive	19.12	Yes	261	Yes	Atezo-chemo	No	No
p022	Baseline	1.87	Yes	Negative	12.71	No	167	No	Atezo-chemo	No	No
p023	Week 9	5.36	Yes	Positive	15.95		229		Atezo-chemo	No	Yes
p023	Baseline	5.36	Yes	Positive	13.84	No	200	Yes	Atezo-chemo	No	Yes
p024	Week 9	7.36	Yes	Positive	14.44		179		Atezo-chemo	No	Yes
p024	Baseline	7.36	Yes	Positive	12.01	No	153	No	Atezo-chemo	No	Yes
p025	Baseline	1.61	Yes	Negative	19.44	Yes	256	Yes	Placebo-chemo	No	No
p026	Baseline	1.81	Yes	Positive	14.34	Yes	209	Yes	Placebo-chemo	Yes	No
p026	Week 9	1.81	Yes	Positive	15.21		225		Placebo-chemo	Yes	No
p027	Baseline	3.52	Yes	Negative	13.62	No	194	Yes	Placebo-chemo	No	No
p027	Week 9	3.52	Yes	Negative	14.55		203		Placebo-chemo	No	No
p028	Baseline	1.81	Yes	Positive	12.68	No	180	No	Placebo-chemo	No	No
p029	Baseline	5.46	Yes	Positive	10.17	No	132	No	Atezo-chemo	No	Yes
p029	Week 9	5.46	Yes	Positive	8.24		105		Atezo-chemo	No	Yes
p030	Week 9	1.84	Yes	Negative	8.43		97		Placebo-chemo	No	No
p030	Baseline	1.84	Yes	Negative	7.43	No	83	No	Placebo-chemo	No	No
p031	Week 9	3.42	Yes	Negative	16.00		189		Atezo-chemo	No	No
p031	Baseline	3.42	Yes	Negative	13.85	Yes	171	No	Atezo-chemo	No	No
p032	Week 9	1.61	Yes	Negative	11.38		139		Placebo-chemo	No	No
p032	Baseline	1.61	Yes	Negative	16.33	Yes	217	Yes	Placebo-chemo	No	No
p033	Week 9	5.62	Yes	Negative	19.05		259		Placebo-chemo	No	Yes
p033	Baseline	5.62	Yes	Negative	20.71	Yes	290	Yes	Placebo-chemo	No	Yes
p034	Baseline	1.74	Yes	Negative	8.03	No	88	No	Atezo-chemo	No	No
p034	Week 9	1.74	Yes	Negative	9.68		109		Atezo-chemo	No	No
p035	Baseline	1.35	Yes	Positive	10.63	No	125	No	Placebo-chemo	No	No
p036	Baseline	1.81	Yes	Negative	12.44	No	170	No	Atezo-chemo	No	No
p036	Week 9	1.81	Yes	Negative	12.96		162		Atezo-chemo	No	No
p037	Week 9	3.68	Yes	Negative	13.38		150		Atezo-chemo	Yes	Yes
p037	Baseline	3.68	Yes	Negative	14.97	Yes	185	Yes	Atezo-chemo	Yes	Yes
p038	Baseline	25.71	Yes	Negative	10.12	No	125	No	Atezo-chemo	No	Yes
p039	Week 9	3.55	Yes	Positive	8.58		105		Placebo-chemo	No	No
p039	Baseline	3.55	Yes	Positive	10.25	No	148	No	Placebo-chemo	No	No
p040	Week 9	3.88	Yes	Positive	11.57		168		Atezo-chemo	No	Yes
p040	Baseline	3.88	Yes	Positive	12.62	No	159	No	Atezo-chemo	No	Yes
p041	Week 9	5.62	Yes	Negative	10.88		146		Atezo-chemo	Yes	Yes
p041	Baseline	5.62	Yes	Negative	10.46	No	143	No	Atezo-chemo	Yes	Yes
p042	Baseline	9.63	Yes	Positive	15.60	Yes	233	Yes	Atezo-chemo	No	Yes
p042	Week 9	9.63	Yes	Positive	14.65		205		Atezo-chemo	No	Yes
p043	Baseline	2.63	Yes	Missing	16.19	Yes	220	Yes	Placebo-chemo	No	Yes
p044	Baseline	1.55	Yes	Negative	10.21	No	129	No	Placebo-chemo	No	No
p045	Baseline	8.98	Yes	Positive	16.35	Yes	204	Yes	Atezo-chemo	No	Yes
p045	Week 9	8.98	Yes	Positive	16.45		218		Atezo-chemo	No	Yes
p046	Week 9	3.72	Yes	Negative	9.18		117		Atezo-chemo	Yes	No
p046	Baseline	3.72	Yes	Negative	13.84	Yes	184	Yes	Atezo-chemo	Yes	No
p047	Baseline	1.15	Yes	Positive	13.11	No	194	Yes	Atezo-chemo	No	No
p048	Week 9	3.52	Yes	Negative	7.79		67		Atezo-chemo	Yes	No
p048	Baseline	3.52	Yes	Negative	13.77	No	175	No	Atezo-chemo	Yes	No
p049	Week 9	1.58	Yes	Positive	17.27		236		Atezo-chemo	No	No
p049	Baseline	1.58	Yes	Positive	17.96	Yes	226	Yes	Atezo-chemo	No	No
p050	Baseline	8.71	Yes	Positive	14.28	Yes	207	Yes	Atezo-chemo	Yes	Yes
p050	Week 9	8.71	Yes	Positive	13.38		200		Atezo-chemo	Yes	Yes
p051	Week 9	5.06	Yes	Positive	15.77		210		Atezo-chemo	No	No
p051	Baseline	5.06	Yes	Positive	17.27	Yes	223	Yes	Atezo-chemo	No	No
p052	Week 9	11.11	Yes	Positive	15.21		191		Placebo-chemo	No	Yes
p052	Baseline	11.11	Yes	Positive	15.95	Yes	215	Yes	Placebo-chemo	No	Yes
p053	Baseline	6.48	Yes	Negative	19.51	Yes	281	Yes	Placebo-chemo	Yes	Yes
p053	Week 9	6.48	Yes	Negative	18.32		269		Placebo-chemo	Yes	Yes
p054	Baseline	11.41	Yes	Positive	15.32	Yes	199	Yes	Atezo-chemo	Yes	Yes
p054	Week 9	11.41	Yes	Positive	15.30		206		Atezo-chemo	Yes	Yes
p055	Baseline	5.69	No	Negative	18.26	Yes	258	Yes	Atezo-chemo	No	Yes
p055	Week 9	5.69	No	Negative	17.20		242		Atezo-chemo	No	Yes
p056	Week 9	8.09	Yes	Positive	11.90		167		Placebo-chemo	No	Yes
p056	Baseline	8.09	Yes	Positive	11.01	No	147	No	Placebo-chemo	No	Yes
p057	Week 9	3.85	Yes	Negative	12.13		143		Atezo-chemo	No	Yes
p057	Baseline	3.85	Yes	Negative	13.40	No	156	No	Atezo-chemo	No	Yes
p058	Week 9	0.03	No	Positive	13.16		181		Placebo-chemo	No	No
p058	Baseline	0.03	No	Positive	11.87	No	156	No	Placebo-chemo	No	No
p059	Week 9	1.87	Yes	Positive	3.92		43		Atezo-chemo	No	No
p059	Baseline	1.87	Yes	Positive	5.72	No	71	No	Atezo-chemo	No	No

1 Patients have been pseudonymized
2 Progression-free survival in months
3 Faith's PD considered as a continous variable
4 High Faith's PD defined as Faith's PD above the median of Faith's PD in baseline samples
5 Observed ASVs considered as a continous variable
6 High observed ASVs defined as observed ASVs above the median of observed ASVs in baseline samples
7 Immune related adverse events of any grade
