# Supplementary material for: Gut microbiota diversity is prognostic and associated with benefit from chemo‐immunotherapy in metastatic triple‐negative breast cancer
Source: Mol Oncol. 2024 Nov 15;19(4):1229–43. doi: 10.1002/1878-0261.13760 (PMC11977656; doi:10.1002/1878-0261.13760)
Supplement: Supplementary file 4 — Table S3. Univariate Cox regression model for progression‐free survival. [file MOL2-19-1229-s001.pdf]

**Characteristic**	**N**	**HR**	**95% CI**	***P* value**
Alpha diversity (binary)	59			
Low		—	—	
High		0.48	0.27-0.85	0.011
Alpha diversity (continuous)	59	0.45	0.24-0.86	0.016
BMI	58	1.02	0.97-1.06	0.5
Age	59	0.97	0.95-0.99	0.003
ECOG	59			
0		—	—	
1		1.19	0.65-2.16	0.6
PD-L1 status	58			
Negative		—	—	
Positive		0.92	0.53-1.59	0.8
De novo metastatic disease	59			
No		—	—	
Yes		0.94	0.52-1.71	0.8
Previous chemotherapy in metastatic setting	59			
Yes		—	—	
No		0.74	0.43-1.29	0.3
Liver metastases	59			
No		—	—	
Yes		1.73	0.99-3.02	0.054
Bone metastases	59			
Yes		—	—	
No		0.7	0.40-1.22	0.2
Previous adjuvant chemotherapy	59			
Yes		—	—	
No		0.69	0.39-1.24	0.2
Any previous chemotherapy	59			
Yes		—	—	
No		0.71	0.37-1.35	0.3
Metastatic sites	59			
≤2		—	—	
>2		2.05	1.15-3.63	0.014

Table S3. Univariate Cox regression model for progression-free survival

Univariate Cox proportional hazards analyses in all patients. Alpha diversity (Faith's PD) was analyzed as both a binary and a continuous variable. Low and high diversity groups of alpha diversity was based on the optimal cutoff score of Faith's PD. Alpha diversity as a continuous variable has been scaled by dividing each value by half of the range of Faith's PD.
