# Supplementary material for: Gut microbiota diversity is prognostic and associated with benefit from chemo‐immunotherapy in metastatic triple‐negative breast cancer
Source: Mol Oncol. 2024 Nov 15;19(4):1229–43. doi: 10.1002/1878-0261.13760 (PMC11977656; doi:10.1002/1878-0261.13760)
Supplement: Supplementary file 5 — Table S4. Multivariate Cox regression model for progression‐free survival. [file MOL2-19-1229-s002.pdf]

Alpha diversity as binary variable			Alpha diversity as continuous variable		
Covariate	HR (95 % CI)	*P* value	Covariate	HR (95 % CI)	*P* value
High Faith's PD (unadjusted)	0.48 (0.27-0.85)	0.011	Faith's PD (unadjusted)	0.45 (0.24-0.86)	0.016
High Faith's PD + age	0.55 (0.31-0.98)	0.041	Faith's PD + age	0.55 (0.28-1.05)	0.071
High Faith's PD + liver metastases	0.54 (0.29-1.00)	0.051	Faith's PD + liver metastases	0.52 (0.26-1.03)	0.060
High Faith's PD + metastatic sites	0.47 (0.27-0.83)	0.009	Faith's PD + metastatic sites	0.42 (0.22-0.79)	0.007

Table S4. Multivariate Cox regression model for progression-free survival

The hazard ratio (HR) and *P* value of progression-free survival for Faith's PD is shown unadjusted and after adjusting for selected variables. Faith's PD is treated as both a binary variable (based on the optimal cutoff value) and as a continuous variable. Age is used as a continuous variable, liver metastases and metastatic sites are categorical. Faith's PD as a continuous variable has been scaled by dividing each value by half of the range of Faith's PD.
